# Decoding Complex Chemical Mixtures with a Physical Model of a Sensor Array

**DOI:** 10.1371/journal.pcbi.1002224

**Published:** 2011-10-20

**Authors:** Julia Tsitron, Addison D. Ault, James R. Broach, Alexandre V. Morozov

**Affiliations:** 1 Department of Physics & Astronomy and BioMaPS Institute for Quantitative Biology, Rutgers University, Piscataway, New Jersey, United States of America; 2 Department of Molecular Biology, Princeton University, Princeton, New Jersey, United States of America; University of Illinois at Urbana-Champaign, United States of America

## Abstract

Combinatorial sensor arrays, such as the olfactory system, can detect a large number of analytes using a relatively small number of receptors. However, the complex pattern of receptor responses to even a single analyte, coupled with the non-linearity of responses to mixtures of analytes, makes quantitative prediction of compound concentrations in a mixture a challenging task. Here we develop a physical model that explicitly takes receptor-ligand interactions into account, and apply it to infer concentrations of highly related sugar nucleotides from the output of four engineered G-protein-coupled receptors. We also derive design principles that enable accurate mixture discrimination with cross-specific sensor arrays. The optimal sensor parameters exhibit relatively weak dependence on component concentrations, making a single designed array useful for analyzing a sizable range of mixtures. The maximum number of mixture components that can be successfully discriminated is twice the number of sensors in the array. Finally, antagonistic receptor responses, well-known to play an important role in natural olfactory systems, prove to be essential for the accurate prediction of component concentrations.

## Author Summary

Mammalian and insect olfactory systems are combinatorial in nature - instead of activating a single specialized receptor, each analyte invokes a complex pattern of responses across the receptor array. The advantage of such systems lies in their ability to detect a large number of analytes with a relatively small number of receptors. However, the complexity of array responses to mixtures of analytes makes quantitative prediction of component concentrations a challenging task. Here we show that combinatorial output from an array of four engineered G-protein-coupled receptors can be used to predict the concentration of each component in mixtures of highly related sugar nucleotides. We employ a physical model of ligand-receptor interactions and carry out Bayesian analysis of the array output. Furthermore, our *in silico* designs of receptor arrays reveal that antagonistic responses, in which the receptor is bound by the ligand but there is no downstream reporter activity, are necessary for precise recognition of mixture components. This conclusion provides a rationale for the widespread inhibitory responses observed in olfactory systems. Our methodology can be employed with both biological systems and artificial receptor arrays (“electronic noses”) designed for various industrial needs.

## Introduction

Mammalian and insect olfactory systems are capable of recognizing tens of thousands of odors – mostly organic compounds with diverse chemical structures and properties [1]–[4]. The olfactory tasks commonly faced by such systems include detecting odors, estimating their strength, identifying their source, and recognizing one specific odor in the background of another [5]. The sense of smell exhibits amazing sensitivity and discriminatory power, distinguishing between closely related compounds and detecting vanishingly small odorant concentrations [6]. Olfactory signaling is mediated by a superfamily of several hundred G-protein-coupled receptors (GPCRs) – a significant fraction of the total number of genes in many higher eukaryotes [7]–[11]. In mammals, GPCRs are located on the surfaces of the cilia projected from olfactory sensory neurons; typically receptors of only one type are expressed in a given neuron [12]. Odor recognition is combinatorial, with one odorant activating multiple receptors and one receptor responding to multiple odorants [12]–[16]. The resulting complex patterns of receptor activation enable robust identification of many more odors than would have been possible with “lock and key” receptors reacting to only one analyte. Moreover, several studies provide evidence for widespread inhibitory responses in which receptors are antagonized by odorants [14], [15], [17]–[20].

The idea of combinatorial recognition has been adapted to artificial arrays in which multiple sensors with partially overlapping selectivities respond to a given analyte [21]–[24]. While the output of these cross-specific arrays in response to single compounds can generally be interpreted through pattern recognition algorithms [24]–[27], computational analysis becomes more difficult when the array is presented with a mixture of compounds. Indeed, the non-linear nature of sensor responses to multiple ligands makes it hard to train discriminatory algorithms on a “typical” subset of patterns. The non-linear dependence of sensor output on ligand concentrations is generic in reporter systems and may be compounded by potential binding interference of the two ligands, saturation of the sensor output [28] and, of particular concern, potential antagonistic action of one ligand on another's activity [19]. As a result, responses to complex mixtures have primarily been used to “fingerprint” specific mixtures rather than identify their constituents quantitatively [29]–[32]. There are relatively few studies which focus on the quantitative analysis of mixtures: for example, Heilig et al. used a single sensor and Fourier transformation techniques to analyze a binary mixture of 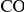 and 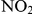 [33], White et al. trained artificial neural networks to identify relative concentrations in binary mixtures [34], and Woodka et al. used a non-negative least squares method to quantify the composition of analyte mixtures with up to five components [35].

Here we describe a physical model of receptor-ligand recognition that explicitly relates observed response patterns to component concentrations and receptor properties, making it easier to quantify mixture constituents. We use Bayesian inference to predict absolute concentrations of each ligand in arbitrary mixtures of uridine diphosphate (UDP) sugar nucleotides applied to a combinatorial array of four GPCRs. Furthermore, we develop a universal metric of receptor array performance, and use it to study the fundamental limits imposed on the accuracy of ligand recognition by the physics and biology of receptor-ligand interactions. Finally, we provide design guidelines for constructing cross-specific arrays optimized for mixture recognition, and demonstrate that inhibitory responses are essential for simultaneous detection of all components in a complex mixture.

## Results

### Biological implementation of the sensor array

Our sensor array is comprised of four engineered receptors (L-3, H-20, K-3 and 2211) with distinct but overlapping specificities for four types of nucleotide sugars: UDP-glucose (UDP-Glc), UDP-galactose (UDP-Gal), UDP-glucosamine (UDP-GlcNAc) and UDP. The receptors were evolved *in vitro* from the human UDP-glucose receptor using directed mutagenesis of the residues involved in ligand binding (see **Materials and Methods**) [36]. Nucleotide sugars and their derivatives are key constituents in polysaccharide synthesis and other cellular processes. Their structural similarity makes them a challenging target for array-based discriminatory analysis. To assess receptor-ligand interactions quantitatively in our sensor array, we functionally expressed the receptors in *S.cerevisiae*. To do so, we replaced the yeast pheromone receptor with one of the sensor GPCRs in strains in which the pheromone response pathway was modified to respond to the heterologous receptor by inducing transcription of the *E. coli* lacZ gene [37]. In this fashion, the extent of GPCR activation following ligand addition could be directly monitored as the level of 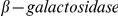 produced in the cell, which we measured using a fluorescence-based assay (**Materials and Methods**). Applying a mixture of nucleotide sugars to the receptor array yields a complex pattern of responses of the four receptor-bearing strains. The response of each receptor depends on the concentration of all components in the mixture, on the receptor-ligand binding affinities, and on the efficacy with which each ligand activates the receptor. Nonetheless, the contents of arbitrary nucleotide sugar mixtures can be deciphered using array readout as input to a physical model of receptor-ligand interactions.

### Physical model of the sensor array

#### Single-receptor, single-ligand model

**Figure 1:**
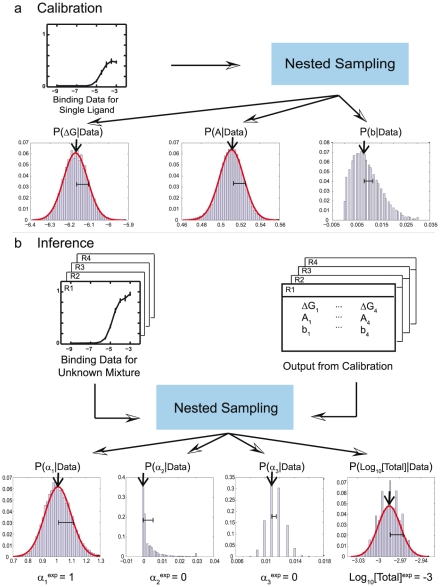
Bayesian algorithm for predicting ligand concentrations in mixtures. (**a**) Calibration of the algorithm: single-ligand, single-receptor binding curves are used to infer binding free energy 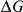, efficacy 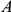 and background intensity 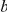 for every receptor-ligand combination. Histograms for each predicted parameter are based on an ensemble of 50000 models sampled by Metropolis Monte Carlo [42] starting from the log-likelighood maximum found by nested sampling [38]. Arrows and error bars indicate the most likely value of each parameter and its standard deviation. (**b**) Inference of ligand concentrations in an unknown mixture. Model parameters from (**a**) together with the response curves for all receptors serve as input to the nested sampling algorithm which predicts relative concentrations 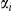 for each component (with respect to one arbitrarily chosen component, cf. Eq. (7)) and the total concentration 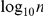 of all ligands in the mixture. Together these predictions yield absolute concentrations for each constituent ligand. Histograms, arrows and error bars have the same meaning as in (**a**), and experimental values are shown below each panel ([Total] = 1 mM at the reference point). For each binding curve, intensity normalized by the maximum intensity on the plate is plotted against 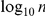 (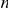 is the total ligand concentration in M).

We start with the simplest case in which a receptor interacts with a single ligand. We assume that the observed signal in our receptor-bearing reporter strain is proportional to the probability that the receptor is bound by the ligand. This proportionality value, 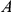, which we refer to as the receptor efficacy, can range from 1, for a full agonist, to 0, for a full antagonist. Thus, for a single receptor interacting with a single ligand, the amount of activation of the reporter in the receptor-bearing strain is given by Eq. (1) (**Materials and Methods**). Reporter activation measurements as a function of single ligand concentration are shown in **Fig. S1a**. We use these data to estimate the parameters of Eq. (1) (**Fig. 1a**) and the amount of experimental noise 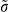 for each single-receptor, single-ligand combination using Bayesian inference with nested sampling [38] (**Materials and Methods**). The most likely values of the parameters (**Table S1**) are then used for subsequent evaluation of mixtures of compounds. The accuracy of parameter predictions depends on the range of concentrations available for these calibration experiments (**Fig. S2**) and on the amount of experimental noise (**Fig. S3**).

#### Multiple-receptor, multiple-ligand model

Once all receptor-ligand interaction parameters have been determined through the analysis of single-ligand calibration experiments, we can proceed to interrogating mixtures of ligands with receptor arrays. In considering the response of receptor-bearing strains to ligand mixtures, we note that each ligand contributes to the overall receptor occupancy and that each receptor molecule on the cell surface activates the reporter with an efficacy specified by the ligand to which it is bound, which is often different for different ligands (**Table S1**). Assuming that all ligands bind competitively to the same site on the receptor, we model the response of the receptor-bearing strain to mixtures of compounds by calculating the total intensity as a sum of fractional occupancies of the receptor by each ligand weighted by the corresponding efficacies (Eq. (6)). We treat each of the receptor-bearing strains with an unknown mixture, sequentially diluted to provide a series of samples across a million-fold range of concentrations (**Fig. S1b**). We carry out Bayesian inference for the entire receptor array, predicting the total concentration of all ligands and the concentration ratios of ligand pairs (**Fig. 1b**). From these values we can deduce the absolute concentration of each ligand in the mixture.

#### Tests of the physical model of mixture recognition

**Figure 2:**
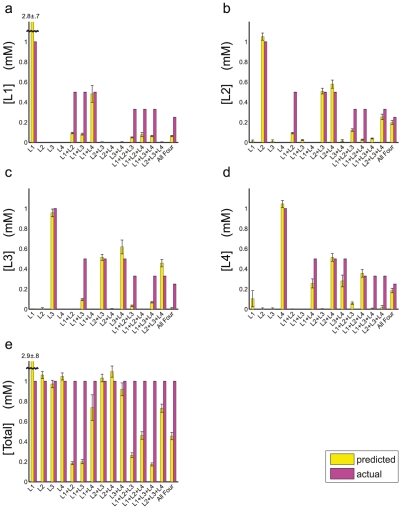
Prediction of ligand concentrations in equal-proportion mixtures. We used nested sampling of a four-receptor, four-ligand model to estimate means and standard deviations for the relative concentrations of all ligands in the mixture and the total ligand concentration at the 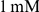 reference point (see **Materials and Methods**). These predictions were converted into absolute concentrations (mM) for each ligand at the 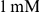 reference point. L1: UDP, L2: UDP-Gal, L3: UDP-Glc, L4: UDP-GlcNAc.

We have tested our approach using a series of assays in which a known combination of ligands was applied to the receptor-bearing strains. As an initial test, we mixed equal proportions of two, three and four ligands in all possible combinations and predicted absolute ligand concentrations. We used a model in which four ligands interacted with four receptors, even if only one, two or three ligands were actually present in the mixture. As can be seen in **Fig. 2** and **Table S2**, our approach is generally quite successful in identifying both zero and non-zero ligand concentrations in the mixtures. For example, with single ligands and binary mixtures the correct chemical or pair of chemicals is predicted to have the highest concentrations in all 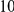 cases. However, the inference is consistently less accurate with UDP-containing mixtures, due in part to larger errors in the predicted total concentration. Thus UDP-related efficacies and binding free energies are less optimal than those of other ligands, as will be demonstrated in detail below.

**Figure 3:**
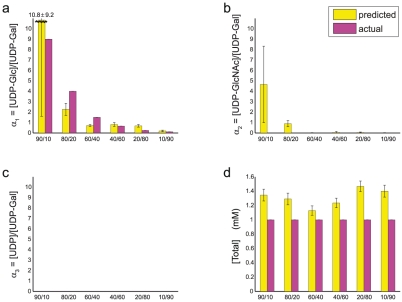
Prediction of ligand concentrations in unequal-proportion binary mixtures of [UDP-Gal] and [UDP-Glc]. We used nested sampling of a four-receptor, four-ligand model to estimate means and standard deviations for the relative concentrations 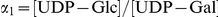, 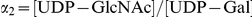, 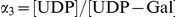 and the total concentration (M) [Total] = [UDP-Gal]+[UDP-Glc]+[UDP-GlcNAc]+[UDP] at the 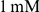 reference point (see **Materials and Methods**). We found that our predictions were improved if 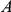
's and 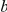
's were refit to account for “plate bias” (cf. header of **Table S4**): small deviations in the values of 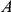 and 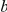 (from the standard values shown in **Table S1** and used everywhere else) between measurements 1–3 (Plate 1) and 4–6 (Plate 2).

Our second test involved combining UDP-Glc and UDP-Gal in several unequal proportions and applying the resulting mixture to the four-receptor array (**Fig. 3**, **Table S3**). As before, we use a four-ligand model, which should predict zero concentrations for UDP-GlcNAc and UDP. The predicted values of 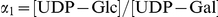 show that the ratio of [UDP-Glc] to [UDP-Gal] is successfully ranked in all cases except for the 60/40 and 40/60 mixtures. Apart from the excessive values of 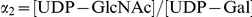 in the 90/10 and 80/20 cases, which are nonetheless not as large as 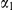, concentrations of all ligands absent from the mixture are correctly inferred to be close to zero. We obtain similar results with the alternative definition of 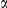
's (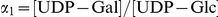, etc.) (**Table S4**), showing that our approach is not overly sensitive to the arbitrary definition of relative concentrations.

**Figure 4:**
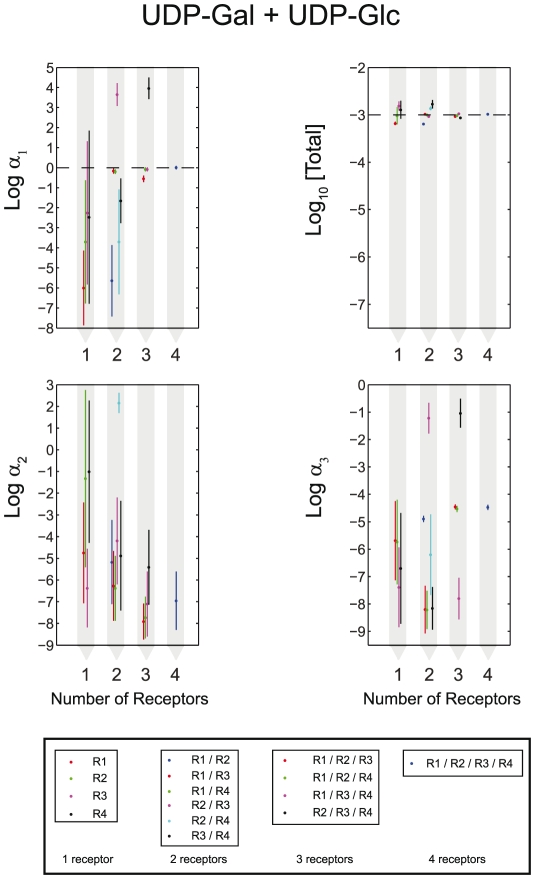
Inference of ligand concentrations is improved with the number of receptors interrogating the mixture. Shown on the log-scale are means and standard deviations for 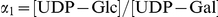, [Total] = [UDP-Glc]+[UDP-GlcNAc]+[UDP-Gal]+[UDP], 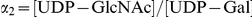, and 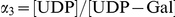. The data are for the 50-50 [UDP-Glc]-[UDP-Gal] binary mixture, leading to 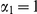, 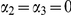, and 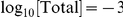 at the reference point. The means and standard deviations were predicted by nested sampling using the four-ligand model and up to four receptors: H-20 (R1), K-3 (R2), L-3 (R3), 2211 (R4).

Increasing the number of receptors should improve prediction accuracy by providing additional information about the mixture. To see the extent of these improvements, we have used a variable number of receptors to infer component concentrations in six equal-proportion mixtures of two nucleotide sugars from **Fig. 2** (**Fig. 4**, **Fig. S4**). As expected, the errors rapidly get smaller as the number of receptors is increased, making larger arrays unnecessary. Surprisingly, in several cases adding extra receptors makes the errors somewhat worse before they become better again (see e.g. the R3 and R3/R4 error bars in the UDP+UDP-Gal 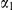 panel of **Fig. S4**), indicating that the noise in the new data outweighs the benefit of additional measurements.

As evident from the activation profile of each receptor in response to each ligand (**Fig. S1a**), the receptors differ from each other in fairly subtle ways. In particular, different ligands do not invoke markedly orthogonal profiles of receptor responses. Nonetheless, even with this suboptimal array design, our algorithm provides accurate identification of ligands present in a mixture and a reasonable assessment of the relative amounts of each.

### Optimization of sensor array performance

#### Hessian analysis of sensor arrays

Our Bayesian approach estimates posterior probabilities for the concentration of each component in an arbitrary mixture. With sufficient data, variation of the posterior probability with model parameters is determined by the corresponding log-likelihood (Eq. (8)), which can be visualized as a multidimensional landscape. The global maximum on this landscape corresponds to the model that best describes the data, while the curvature at the maximum shows how sensitive the likelihood is to the change in each parameter. Narrow peaks result in precisely defined parameter values, whereas wide plateaus yield many nearly equivalent predictions and therefore sizable uncertainties in parameter estimates. Expanding the log-likelihood in the vicinity of its maximum yields a Hessian matrix (Eq. (9)), which contains information about standard deviation 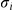 of each model parameter 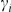 (Eq. (10)) [39]. For example, if the observed receptor response does not depend on 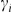, zero entries appear in the Hessian, leading to the infinite uncertainty 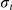. Making all Hessian matrix elements uniformly larger leads to the smaller 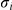 for each predicted parameter 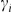.

Hessian analysis relies on the quadratic expansion in the vicinity of the log-likelihood maximum and hence it is important to check how well it captures the behavior of the more general but computationally intensive nested sampling approach. To create a test case for which the answer is known, we have used Eq. (6) to generate synthetic data for 15 equal-proportion mixtures from **Fig. 2** in the low-noise limit (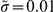 for all receptors, several times smaller than experimental values from **Table S1**). We observe close correspondence between parameter uncertainties inferred from nested sampling vs. Hessian analysis (**Fig. S5**). Moreover, since larger uncertainties make it easier for the average values of predicted parameters to be incorrect, there is also correlation between Hessian errors and the absolute differences between mean predicted and true values (**Fig. S6**). The Hessian-based approach remains useful when experimental data, for which the precise model is unknown and the noise is substantially higher (**Table S1**), is analyzed in the same way: the average over 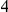 correlation coefficients between Hessian errors and standard deviations from nested sampling (computed for 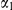, 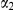, 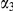 and 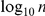) is 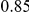, and the average over 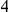 correlation coefficients between Hessian errors and absolute differences between predicted and true values is 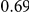. In both real and synthetic cases, the Hessian matrix was computed with correct relative and total concentrations and 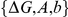 values from **Table S1**. We conclude that Hessian errors are a reasonable measure of sensor array performance.

Not all receptors are equally good candidates for inclusion into biosensor arrays – for example, receptors with similar sets of efficacies and binding affinities should be less useful than receptors with more orthogonal binding and activation patterns. Here we make such qualitative insights precise by developing a Hessian approach to biosensor array design. That is, given a certain number of measurements with an array of fixed size (typically, a series in which the total concentration is changed step-by-step within a certain range), we wish to derive the most optimal choice of receptor properties for deciphering the mixture. From the Hessian point of view, the best array will have the smallest errors in predicting component concentrations (Eq. (10)). Because each error is inversely proportional to the determinant of the Hessian, we maximize the determinant instead of minimizing the errors directly. Similarly to the prediction of constituent concentrations, the maximization is carried out by nested sampling [38]. In general, the most optimal receptor parameters and their robustness will depend on the relative concentration of each component in a mixture and on the number of measurements made with the array. For example, an array fine-tuned to detect small admixtures of compound B in the background of compound A may function less well if the concentrations of A and B become approximately equal.

#### Optimal parameters for a single receptor interacting with a two-ligand mixture

**Figure 5:**
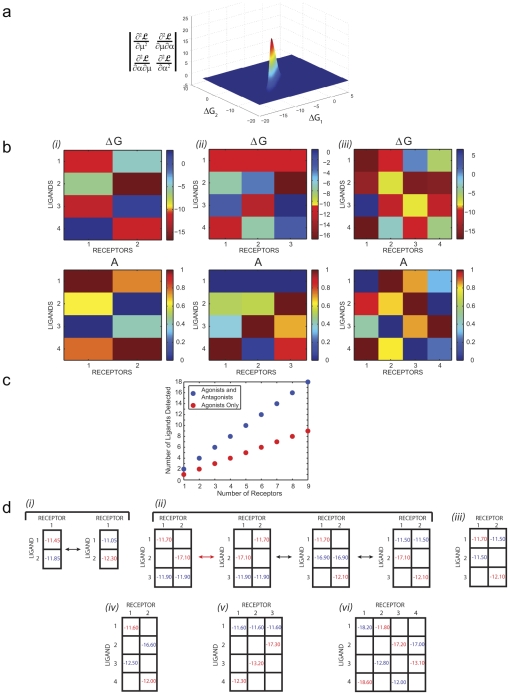
Optimal design of receptor arrays. (**a**) Determinant of the Hessian in the one-receptor, two-ligand case, plotted as a function of binding energies 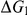 and 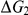. The binding energies at the peak are 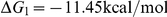 and 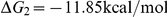. The efficacies are fixed at 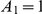, 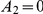; 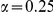. (**b**) Optimal free energies 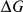 and efficacies 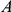 obtained by maximizing the determinant of the Hessian in the (*i*) two-receptor, four-ligand, (*ii*) three-receptor, four-ligand and (*iii*) four-receptor, four-ligand cases. 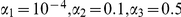. (**c**) The number of successfully discriminated ligands increases linearly with the number of receptors: 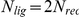 if the determinant of the Hessian is maximized with respect to all binding energies 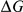 and efficacies 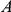 (separately for each 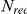); 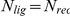 if all ligands are forced to be full agonists with unit efficacies. We call all ligands successfully discriminated if 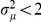, 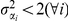 in a given nested sampling run. Two alternative choices of relative concentrations: 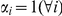 and 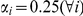 yielded the same linear dependence on the number of receptors. (**d**) Optimal 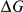 values are shown for several cases: (*i*) one-receptor, two-ligand, (*ii*) two-receptor, three-ligand, (*iii*) two-receptor, three-ligand (at a local maximum), (*iv*) two-receptor, four-ligand, (*v*) three-receptor, four-ligand and (*vi*) four-receptor, four-ligand. Values in blue correspond to an efficacy of 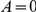 (full antagonist), while values in red correspond to 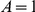 (full agonist). 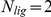: 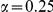; 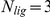: 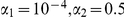; 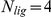: 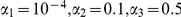. In all cases shown in (**a**)–(**d**), we used 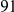 datapoints for each receptor (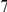 replicates with 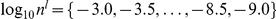).

To demonstrate our approach, we first optimize parameters of a single receptor discriminating a mixture of two ligands. By maximizing the determinant of the Hessian, in this case a 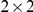 matrix, as a function of two efficacies and two binding energies, we find that the best discrimination is achieved if one ligand acts as an agonist and the other as an antagonist: 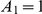 and 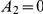 *or* 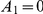 and 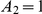 (for simplicity, background intensities were set to 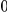 in all sensor array designs). Although in both cases each ligand binds strongly to the receptor, there is a unique set of optimal binding energies 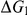 and 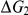 for each agonist-antagonist scenario (**Fig. 5a**, **Fig. S7**). The actual values of the binding energies depend on the relative concentration 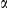; for unequal ligand concentrations the two 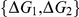 sets will in general be distinct. This is not surprising since exchanging ligand labels amounts to exchanging relative concentrations of the agonist and the antagonist in the mixture. The height of the peak in both determinant landscapes is the same, indicating that the two alternative solutions lead to equally acceptable array designs as long as the 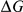
's are tuned appropriately.

The fine-tuning of binding energies is not necessary if either the total concentration 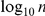 is known and the task is to minimize the error in predicting the relative concentration 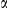, or vice versa (**Fig. S7** and **S8**). The single-peak landscape structure appears only if the absolute concentrations of both components need to be predicted together. Strikingly, simultaneous prediction of the total and relative concentrations is impossible with the agonist-agonist receptor response (**Fig. S9**, **Text S1**). The dependence of the optimal binding energies on the value of 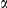 is fairly weak (**Fig. S10**). Thus one set of 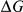
's optimized for a specific value of 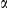 provides a near-optimal solution for a sizable range of ligand concentrations.

#### Design of multiple-receptor, multiple-ligand arrays

The agonist-antagonist pattern observed in the one-receptor, two-ligand case plays the role of a basic building block when two or more receptors interact with multiple ligands: nested sampling maximization of the Hessian determinant with respect to binding energies 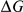 and efficacies 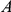 reveals that the array as a whole performs best if each receptor binds one agonist and one antagonist. For example, in the two-receptor, four-ligand case (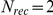, ) receptor 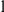 strongly binds ligands 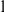 and 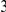 with 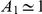 and 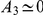, whereas receptor 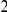 strongly binds ligands 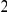 and 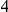 with 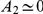 and 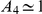 (**Fig. 5b** (***i***)). Each ligand preferentially binds to only one receptor. When another receptor is added to the array, the optimal binding and activation pattern becomes strikingly different: each receptor once again binds both an agonist and an antagonist but ligand 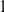 now acts as an antagonist to all three receptors (**Fig. 5b **(***ii***)). Each of the other three ligands is an agonist to one of the receptors. In the 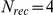, 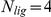 case each ligand is an agonist for one receptor and an antagonist for another (**Fig. 5b **(***iii***)). Once again, each receptor binds both an agonist and an antagonist. The determinant of the Hessian is dominated by these agonist-antagonist patterns, and is less sensitive to the changes in efficacies and binding energies that do not affect them.

In the light of the observed agonist-antagonist behavior, it is not surprising to see that each receptor can identify concentrations of at most two ligands (**Fig. 5c**, blue dots). The uncertainty in predicting components of the mixture is minimized if for every receptor one ligand binds strongly as a full agonist and another as a full antagonist. As we have seen, when receptor parameters are less than optimal, the discrimination is still possible but additional receptors may be required: three or four rather than two in the four-ligand case (**Fig. 4**, **Fig. S4**). If we eliminate the agonist-antagonist degree of freedom by setting all efficacies to 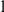, discriminating 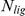 requires twice as many receptors (**Fig. 5c**, red dots). In this case each receptor is strongly bound by only one ligand, measuring its concentration independently of the other members of the array. Having access to the full range of receptor responses makes it possible to double the number of ligands in the mixture, but the relationship between 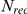 and 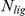 remains linear.

#### Symmetry properties of optimal sensor arrays

The patterns shown in **Fig. 5b** are not unique – indeed, alternative agonist-antagonist patterns can be generated simply by exchanging receptor labels. Less trivially, a given ligand can be an agonist or an antagonist for different combinations of receptors. In the simplest case of one receptor interacting with two ligands, this symmetry generates two equivalent global maxima discussed above: 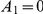, 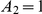 and 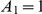, 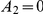 (**Fig. 5d **(***i***)). In the two-receptor, three-ligand case symmetry arguments combined with extensive sampling yield three global maxima of the Hessian determinant. Each global maximum corresponds to the situation where one of the three ligands acts as an antagonist to both receptors (**Fig. 5d **(***ii***)). The red arrow in **Fig. 5d **(***ii***) indicates a trivial exchange of receptor labels, whereas the black arrows connect three different globally optimal solutions. In addition, there are 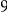 local maxima with one of the ligands acting either as an agonist to both receptors, or as an agonist to one receptor and an antagonist to the other (e.g. **Fig. 5d **(***iii***); see **Text S1** for a complete enumeration).

In general, 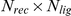 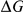
's are necessary to characterize all the global and local peaks on the Hessian determinant landscape, with 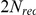 binding energies describing any given agonist-antagonist pattern. The values of the binding energies depend on the component concentrations in the interrogated mixture. In the 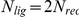 case all maxima are global and each receptor interacts with two unique ligands. To estimate the benefit of additional receptors, we increased the number of receptors from two to three to four in the four-ligand case (**Fig. 5d **(***iv***), ****(***v***), ****(***vi***)). After adding the third receptor the average uncertainty of one total and three relative concentrations, 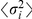, decreased from 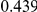 to 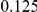. However, only a slight gain was seen when the fourth receptor was added, with 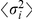 becoming 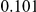. Thus adding more and more receptors to the array yields increasingly marginal improvements after a certain threshold.

The agonist-antagonist rules described above create readout patterns that are not a simple sum of array responses to single-ligand binding. For one receptor optimized to discriminate two ligands (**Fig. 5d **(***i***)), fluorescent response to the mixture is intermediate between full activation by the agonist and full repression by the antagonist (**Fig. S11a**). This intensity modulation provides enough information for decoding the contents of the mixture. Similarly, in the two-receptor, three-ligand case (**Fig. 5d **(***ii***)) a mixture of all three ligands induces a response with intermediate fluorescense levels (**Fig. S11b**). This pattern is distinct from those induced by single ligands and by binary mixtures with the same relative concentrations as in the three-ligand case.

#### Performance analysis and improvement of the experimental biosensor array

The design guidelines described above can be used to predict which parameter changes lead to most significant improvements in performance compared to our currently implemented array. Although we do not have direct experimental control over the values of 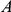 and 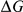, such insights are useful e.g. for choosing the best combination of several receptors from a larger library. Familiar agonist-antagonist patterns emerge when 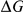
's and 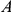
's are optimized either separately or together to discriminate an equal-proportion, four-ligand mixture (**Fig. S12**). In particular, if 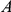
's are kept fixed, 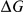
's for the most distant pair of 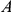
's become more favorable for each receptor, creating an agonist-antagonist pair (**Fig. S12c**). Conversely, if 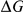
's are fixed, the values of 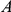 corresponding to the two lowest 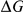
's become more distant from each other (**Fig. S12d**). Not surprisingly, the agonist-antagonist patterns are even more pronounced if both 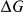
's and 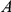
's are allowed to relax (**Fig. S12e** and **S12f**). Because two and certainly three optimized receptors are sufficient for discriminating four-ligand mixtures (**Fig. 5c**), the fourth receptor, which does not follow the usual pattern as strongly as the other three, appears to be superfluous. Similarly to the cases shown in **Fig. 5d**, **Fig. S12e** and **S12f** represent only one solution from a large family of local and global maxima of the Hessian determinant, which are related by permutations of receptor and ligand indices. Optimizing receptor-ligand parameters leads to a sizable improvement in array performance: with 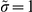 for all receptors, 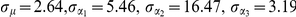 for the original array, whereas 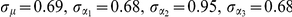 for the array in which both 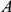
's and 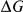
's have been optimized.

For the experimentally implemented four-receptor GPCR array, nested sampling errors are consistently larger when UDP is present in the mixture (**Fig. 2**, **Table S2**). This observation is consistent with Hessian analysis: for example, the average Hessian uncertainties for three UDP-free binary mixtures are 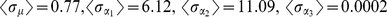. For three UDP-containing binary mixtures, the average Hessian errors are 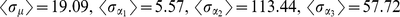 (as before, all Hessian errors are computed with correct concentrations and 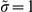 for all receptors). The Hessian determinants are also consistently smaller for UDP-containing binary mixtures. These observations indicate that UDP parameters are further away from the optimal four-receptor array designed to analyze an equal-proportion binary mixture: either 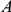
's or 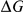
's need to be changed in order to create stronger agonist-antagonist patterns.

## Discussion

We have developed a Bayesian algorithm that allows determination of all the constituents in an unknown mixture from the output of a cross-specific sensor array. Our algorithm employs a physical picture of sensor-analyte interactions to model the non-linear relationship between ligand concentrations and the reporter response. After appropriate calibration of each sensor's response to each analyte of interest, the algorithm interprets the integrated output of the entire array and, with a sufficient number of variably tuned sensors, reliably returns the amount of each chemical in a complex mixture.

We also provide quantitative guidelines for designing optimal sets of sensors. Three general principles emerged from our computational and theoretical studies of array design. First, the optimal parameters of the sensors exhibit weak dependence on the relative amounts of compounds in a mixture. Thus a given set of optimal sensors will remain near-optimal through a sizable range of ligand concentrations. Nonetheless, analyzing a mixture where both compounds are present in roughly similar amounts is better accomplished with a set of sensors different from those fine-tuned to measure a small amount of one compound in the presence of a large excess of the other.

Second, the maximum number of ligands in a mixture whose levels can all be determined simultaneously is simply twice the number of sensors in the array. This linear relationship is different from the exponential relationship between ligands and receptors in olfactory systems [4], [12]. The problem addressed by the olfactory system, to recognize a very large number of individual odors with a limited repertoire of receptors, is not the same as that solved by our algorithm, to determine all the constituents in a complex mixture. In fact, even the most skilled human nose can simultaneously detect and distinguish no more than a handful of odorants.

Third, the optimum design of receptors for the array demands that one of the ligands function as a strong agonist of a receptor and a second ligand as a strong antagonist of that receptor. Antagonists sharpen the discriminatory powers of the array by heightening the differences in the receptor response to individual compounds. As a result, a mixture of chemicals produces an array readout which is not a superposition of responses to individual ligands, and whose intensity pattern may be fine-tuned for maximum recognition through receptor-ligand binding energies. Accordingly, odors that function as antagonists to a subset of olfactory receptors could potentially increase the discriminatory power of the olfactory system, and in particular enable it to resolve mixtures that contain those odors. Recent analysis of olfactory receptors suggests that some odorants do possess antagonist activity [14], [17]–[20]. Our theoretical framework provides a rationale for the existence of such antagonists and underscores their role in both olfactory systems and artificial receptor arrays.

## Materials and Methods

### Targeted mutagenesis and selection of functional receptor mutants

The L-3 mutant was isolated using a procedure similar to that previously employed with the H-20 and K-3 mutants [36]: oligonucleotides with randomized sequences corresponding to the codons to be mutagenized were utilized to generate overlapping PCR products. The L-3 motif corresponds to amino acid residues LLxSA on TM7. Mutant libraries were generated by gap repair using overlapping PCR products and transformed to media selective for recombined plasmids. To select for functional mutants, libraries were replica-plated to selective SC-His media [40] containing one of six ligands: UDP-Gal, UDP-Glc, UDP-galNAc, UDP-GlcNAc, UDP or dTDP-glucose (50 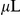 of 1 mM solution spread on 30 mL of SC “Leu-His agar medium in 8.5 cm Petri plates). Yeast growth media was supplemented by 1 mM 3AT, a competitive inhibitor of the *HIS3* reporter gene product, which sets the threshold for reporter gene activation. Functional receptor mutants that showed qualitatively disparate responses to the panel of ligands were selected for further analysis. Among these, the H-20 and K-3 mutants, described earlier [36], and the L-3 mutant, described here, were selected to be utilized alongside the 2211 “parent” in a four-receptor array for analysis of mixtures of UDP-Glc, UDP-Gal, UDP-GlcNAc and UDP.

### 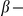
galactosidase assays

Our 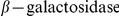 assays were based on microtiter assays described previously [41]. Yeast strains expressing each of the four mutant receptors were diluted to 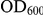 of 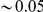 in flasks. Cultures were then grown overnight in 100 mL selective media to an 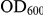 of 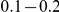. Serial dilutions of each ligand or mixture of ligands were prepared in yeast culture medium in 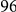-well culture blocks. Ligands or mixtures of ligands were transferred in 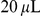 aliquots in quadruplicate to deep-well polypropylene 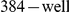 plates using a BioMek robotic liquid handler. 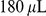 of suspended yeast cells in medium (undiluted from the overnight cultures) were then aliquotted into each well and mixed. The cultures were sealed with foil tape and incubated at 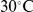 on a plate shaker at 400–500 rpm for 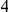 hours (H-20, K-3 and L-3 receptors) or overnight (2211 receptor). After incubation, 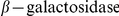 substrate [41] (FDG solution; 0.5 mM fluorescein di-beta-D-galactopyranoside, 2.3% Triton X-100, and 0.127 M Pipes, pH 7.2) was mixed with an equal volume of Pierce Y-PER solution (Thermo Scientific) and distributed in 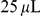 aliquots to black 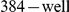 plates. 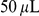 aliquots of the yeast/ligand cultures were then transferred into the black 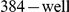 plates and mixed gently but thoroughly by pipetting, taking care to avoid generating bubbles. A single layer of paper towel was placed on top of each plate and the plates were then individually wrapped in aluminum foil and incubated without shaking at 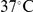 for approximately one hour before reading on an automated fluorescent plate reader (Perkin Elmer EnVision).

Microtiter plate-based assays are often subject to edge- or plate-bias due to uneven heating or discrepancies in timing across a single plate or among plates. While no obvious plate effects were seen, it is very difficult to control for all possible variations in a single experiment. Due to the number of samples and the need to make efficient use of materials, each of the mixture experiments was split across two plates per receptor. In mixtures of equal proportions, samples containing UDP, UDP-Gal and UDP-GlcNAc but lacking UDP-Glc were on Plate 1, while all mixtures containing UDP-Glc were on Plate 2. In the UDP-Gal/UDP-Glc binary mixtures of unequal proportions, samples containing 90%, 80% or 60% UDP-Glc were on Plate 1, while samples containing 40%, 20% or 10% UDP-Glc were on Plate 2.

For each single ligand or combination of ligands, a series of measurements was performed at several values of the total concentration 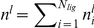 (M): 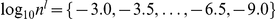 for H-20, K-3, L-3 and 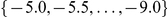 for 2211. The total chemical potential 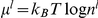 is then given by 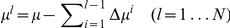, where 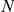 is the number of measurements in the series, 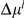 are known chemical potential differences between two consecutive measurements, and 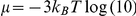 is the chemical potential at the 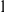 mM reference point. Note that in order to reconstruct the total chemical potential for all points in the series, only 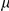 needs to be predicted. Each series of measurements was replicated four times; fluorescence counts were normalized to 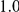 separately for each plate (**Dataset S1**).

### Models of receptor response to ligand binding

For a single receptor interacting with a single ligand, we model the normalized reporter fluorescent intensity as:
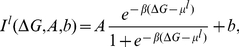
(1)where 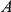 is the receptor efficacy, 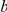 is the background intensity (a small amount of background fluorescence observed in the absence of ligand binding), 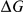 is the free energy of receptor-ligand binding, 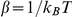 (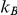 is the Boltzmann constant, and 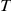 is the temperature), and 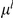 is the chemical potential.

We compute the log-likelihood of the data by assuming that fluorescence measurements are Gaussian-distributed around values from Eq. (1):
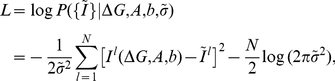
(2)where 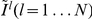 are measured intensities and 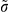 is the noise parameter. The log-likelihood is used to estimate the posterior probability of all model parameters according to the Bayes' formula [38]:
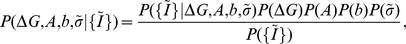
(3)where on the right-hand side the likelihood from Eq. (2) is multiplied by the product of priors for each model parameter and divided by evidence. 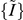 combines data from all experimental replicates. We use uniform priors (invariant with respect to translations, 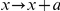):
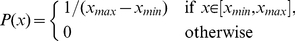
(4)for 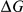, 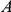 and 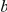, and Jeffrey's priors (invariant with respect to rescaling, 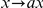) for 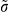:
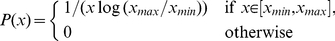
(5)We have used 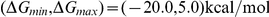, 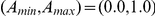, 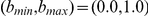, 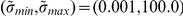 in our calculations.

The reporter response to a mixture of ligands is given by
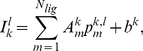
(6)where 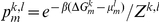 is the probability that receptor 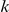 is bound by ligand 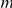 and 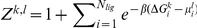 is the partition function. 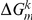 is the binding free energy between receptor 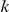 and ligand 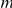 (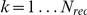, 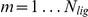), 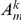 is the efficacy, and 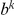 is the background intensity. The background intensity for receptor 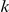 is the average from all calibration experiments involving that receptor. 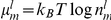 is the chemical potential of ligand 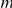, which can be expressed through the total chemical potential 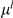 and the relative concentrations 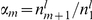 (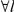, 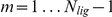):
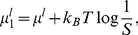
(7)

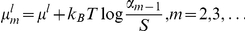
where 
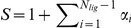
. Note that an arbitrary choice of the ligand in the denominator leads to several equivalent representations of the relative concentrations.

The log-likelihood of the observed pattern of fluorescence intensities from multiple receptors interrogated by a mixture of ligands is given by
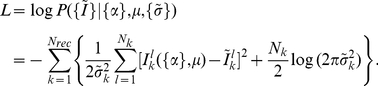
(8)Here 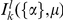 is defined in Eq. (6) (in the interests of brevity, we suppress its dependence on 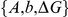 for each receptor-ligand combination). 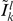 denotes fluorescence measured for receptor 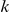 at the total chemical potential 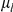, 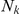 is the total number of measurements, and 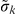 is the noise parameter. Similarly to Eq. (3), the log-likelihood is used to estimate the posterior probability 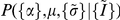. We employ a uniform prior for 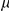 with 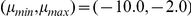 and a Jeffrey's prior for 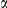
's with 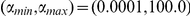.

We estimate all posterior probabilities by nested sampling [38] – a Bayesian Monte-Carlo (MC) technique that yields an ensemble of models from which the average value of each parameter and its standard deviation are computed. Unlike other methods such as MC sampling of the product of likelihood and priors, nested sampling allows us to keep track of the evidence, yielding absolute values of the posterior probability.

### Hessian analysis

The Hessian matrix in the low-noise limit can be written as (**Text S1**):
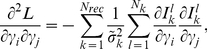
(9)where 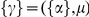, 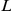 is the log-likelihood, 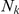 is the total number of measurements for receptor 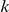, and 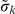 is the noise parameter. Uncertainties 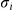 for each predicted parameter 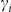 are given by the diagonal elements of the inverse Hessian matrix:
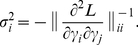
(10)Software used in this study, called RANSA (Receptor Array Nested Sampling Algorithm), is available at http://olfaction.rutgers.edu.

## Supporting Information

Figure S1
**Overview of the GPCR-based biosensor array.** (**a**) Each receptor-ligand combination is tested for functional activation, yielding 16 binding curves. For each curve, intensity normalized by the maximum intensity on the plate is plotted against 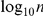 (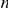 is the ligand concentration in M). The error bars on each curve are from four biological replicates. The single-receptor, single-ligand binding curves are used to calibrate the physical model by inferring 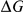, 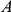 and 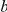 separately for each receptor-ligand combination. (**b**) An unknown mixture of four ligands is applied to each of the four receptors. The resulting fluorescent response curves together with the 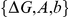 predictions are used as input to the Bayesian algorithm designed to predict absolute concentrations of each ligand in the mixture. R1: H-20, R2: K-3, R3: L-3, R4: 2211.(EPS)

Figure S2
**Prediction accuracy increases with the number of measurements at different total concentrations.** Synthetic data was generated using Eq. (1) in the main text, with 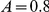, 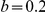, and 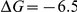 kcal/mol. To account for experimental error, Gaussian noise with 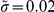 was added to the intensity from Eq. (1). The maximum total concentration of the ligand was gradually increased as shown in the nine panels on top, yielding more and more complete binding curves: 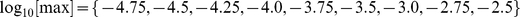. 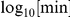 was 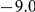 in all cases, and 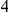 replicates with 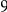 datapoints per curve were created for each concentration range. In each panel 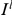 is plotted as a function of 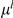 in the absence of noise. For each concentration range, 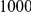 nested sampling runs were carried out to predict 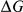, 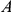 and 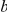. The standard deviation 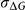 from each run was averaged and plotted in the bottom panel as a function of the total range of ligand concentrations 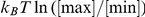. Each dot in the bottom panel is color-coded to correspond to a particular binding curve on top.(EPS)

Figure S3
**Prediction accuracy decreases with the amount of noise in the data.** Synthetic data was generated using Eq. (1) in the main text, with 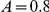, 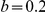, and 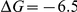 kcal/mol. In analogy with the experiments, we used the concentration range 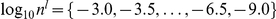 and created 4 replicates, yielding 36 datapoints. To model the increase in experimental error, Gaussian noise with 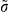 ranging from 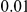 to 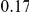 was added to the intensity from Eq. (1). For each value of 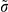, 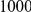 nested sampling runs were carried out to predict 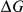, 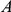 and 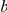. The standard deviation 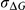 from each run was averaged and plotted as a function of 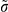.(EPS)

Figure S4
**Inference of ligand concentrations is improved with the number of receptors interrogating the mixture.** Shown on the log-scale are means and standard deviations for three relative concentrations (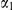, 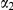, 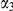) and the total concentration, predicted by nested sampling using the four-ligand model and up to four receptors: H-20 (R1), K-3 (R2), L-3 (R3), 2211 (R4). Each experiment has a 50-50 binary mixture of two ligands indicated on top of each panel, leading to 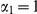, 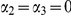, and 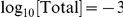 at the reference point ([Total] = [UDP-Glc]+[UDP-GlcNAc]+[UDP-Gal]+[UDP]). **UDP-Gal+UDP-GlcNAc mixture:** 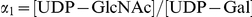, 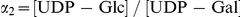, 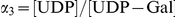. **UDP-Glc+UDP-GlcNAc mixture:** 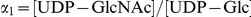, 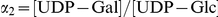, 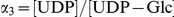. **UDP+UDP-Glc mixture:** 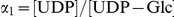, 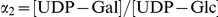, 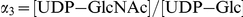. **UDP+UDP-GlcNAc mixture:** 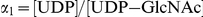, 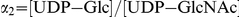, 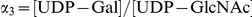. **UDP+UDP-Gal mixture:** 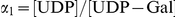, 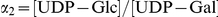, 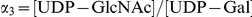.(EPS)

Figure S5
**Hessian uncertainties vs. standard deviations from nested sampling.** Synthetic data was generated as four replicates for each of the 15 equal-proportion mixtures from **Fig. 2**, using parameters from **Table S1** and 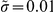 for all receptors (Eq. (6) in the main text). For each receptor, concentration ranges were taken from the corresponding experiment (**Materials and Methods**). For each parameter, a Hessian error computed using Eq. (10) in the main text (x-axis) is compared with the standard deviation from a nested sampling run (y-axis). Nested sampling simultaneously infers relative concentrations 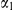, 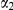, 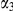, the total concentration and 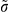
's given receptor-ligand parameters from **Table S1**.(EPS)

Figure S6
**Hessian uncertainties vs. errors in parameter predictions.** Synthetic data was generated as four replicates for each of the 15 equal-proportion mixtures from **Fig. 2**, using parameters from **Table S1** and 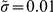 for all receptors (Eq. (6) in the main text). For each receptor, concentration ranges were taken from the corresponding experiment (**Materials and Methods**). For each parameter, a Hessian error computed using Eq. (10) in the main text (x-axis) is compared with the absolute magnitude of the difference between the mean value predicted by nested sampling and the correct value (y-axis). Nested sampling simultaneously infers relative concentrations 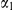, 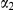, 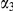, the total concentration and 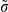
's given receptor-ligand parameters from **Table S1**.(EPS)

Figure S7
**Matrix elements and the determinant of the Hessian in the antagonist-agonist case, plotted as a function of binding energies 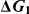 and 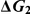 in the one-receptor, two-ligand system.** The efficacies are fixed at 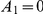, 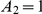; 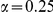. We used 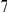 replicates with 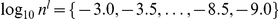. The values of the binding energies at the peak of the determinant landscape are 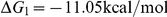 and 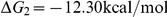.(EPS)

Figure S8
**Matrix elements of the Hessian in the agonist-antagonist case, plotted as a function of binding energies 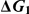 and 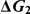 in the one-receptor, two-ligand system.** The efficacies are fixed at 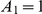, 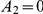; 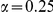. We used 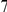 replicates with 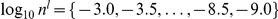.(EPS)

Figure S9
**Matrix elements and the determinant of the Hessian in the agonist-agonist case, plotted as a function of binding energies 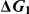 and 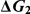 in the one-receptor, two-ligand system.** The efficacies are fixed at 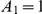, 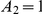; 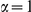. We used 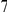 replicates with 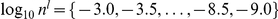.(EPS)

Figure S10
**Changes in the Hessian determinant with the concentration of the second ligand.** Each determinant is plotted as a function of binding energies 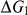 and 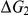 for a given value of 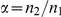. The efficacies are fixed at 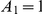, 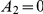. We used 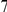 replicates with 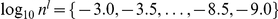. Shown in each panel are the optimal 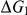 and 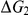 corresponding to the maximum value of the determinant.(EPS)

Figure S11
**Schematic diagram of receptor activation by single ligands and ligand mixtures with optimized binding affinities and efficacies.** (**a**) Two cases of agonist-antagonist single-receptor arrays designed to discriminate a mixture of two ligands (**Fig. 5d **(***i***)). (**b**) Array of two receptors designed to discriminate a mixture of three ligands (**Fig. 5d **(***ii***)). In both cases we show relative intensities (normalized to 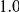) corresponding to 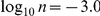. 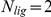: 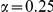; 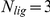: 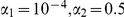 (leading to 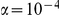, 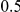 and 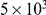 for the binary combinations of ligands 1–2, 2–3 and 1–3, respectively). 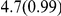 and 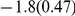 are 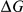
's and 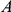
's (in parentheses) for receptor-ligand interactions outside of the dominant agonist-antagonist pattern.(EPS)

Figure S12
**Improving performance of the experimentally implemented sensor array.** Free energies 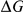 (**a**) and efficacies 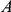 (**b**) in the experimentally implemented sensor array with parameters from **Table S1**. Free energies 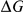 (**c**) and efficacies 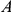 (**d**) from two sensor arrays in which the determinant of the Hessian was maximized only with respect to 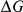
's and 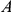
's, respectively. Free energies 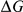 (**e**) and efficacies 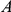 (**f**) in the optimal sensor array in which the determinant of the Hessian was maximized with respect to both 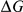
's and 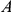
's. The determinant was computed using four replicates of an equal-proportion mixture of four ligands and 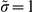 for all receptors. For each receptor, concentrations were taken from the corresponding experiment (**Materials and Methods**). In panels **a**–**d**, the order of ligands is L1: UDP, L2: UDP-Gal, L3: UDP-Glc, L4: UDP-GlcNAc. The order of receptors is R1: H-20, R2: K-3, R3: L-3, R4: 2211. Note that ligand and receptor identities are lost in panels **e**,**f** since all parameters have been optimized.(EPS)

Dataset S1 **Normalized fluorescence intensity measurements from experiments with equal-proportion **(**Fig. 2** **) and unequal-proportion **(**Fig. 3** **) mixtures.**
(XLS)

Table S1
**Parameters of receptor-ligand interactions predicted from one-receptor, one-ligand binding curves (UDP-Gal, UDP-Glc, UDP-GlcNAc) and one-receptor, two-ligand binding curves (UDP).** 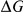 is the receptor-ligand binding free energy (kcal/mol), 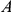 is the receptor efficacy, 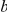 is the background intensity, and 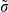 is the noise parameter which quantifies the discrepancy between the model and the observed binding curves. Due to antagonistic activity of UDP, 50/50 UDP+UDP-Glc binary mixture was used with K-3, L-3, 2211 and 50/50 UDP+UDP-Gal binary mixture was used with H-20 to predict UDP parameters (compound concentrations were set to their exact values for these calibration predictions). In each case, the mixture was chosen on the basis of the smallest standard deviation of 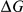.(PDF)

Table S2 **Prediction of ligand concentrations in equal-proportion mixtures (data for** **Fig. 2** **).**
(PDF)

Table S3 **Prediction of ligand concentrations in unequal-proportion binary mixtures of [UDP-Gal] and [UDP-Glc] (data for** **Fig. 3** **).**
(PDF)

Table S4
**Prediction of ligand concentrations in unequal-proportion binary mixtures of [UDP-Gal] and [UDP-Glc] using an alternative definition of relative concentrations.** We used nested sampling of a four-receptor, four-ligand model to infer relative concentrations 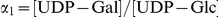, 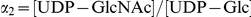, 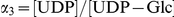, as well as the total concentration [Total] = [UDP-Gal]+[UDP-Glc]+[UDP-GlcNAc]+[UDP] at the 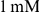 reference point. 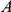
's and 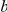
's were refit to account for “plate bias”: small systematic deviations in the values of 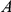 and 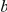 (from the standard values shown in **Table S1** and used everywhere else) between different plates. For Plate 1 (measurements 1–3), 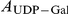, 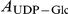 and 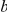 were set to 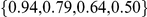, 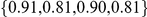 and 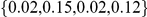 for H-20, K-3, L-3 and 2211, respectively. For Plate 2 (measurements 4–6), the corresponding values were 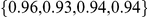, 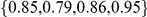 and 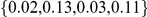. 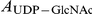 and 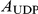 were taken from **Table S1**.(PDF)

Text S1 **Supplementary Materials and Methods.**
(PDF)
