# Supplementary material for: Decoding Complex Chemical Mixtures with a Physical Model of a Sensor Array
Source: PLoS Comput Biol. 2011 Oct 20;7(10):e1002224. doi: 10.1371/journal.pcbi.1002224 (PMC3202980; doi:10.1371/journal.pcbi.1002224)
Supplement: Table S1 — Parameters of receptor-ligand interactions predicted from one-receptor, one-ligand binding curves (UDP-Gal, UDP-Glc, UDP-GlcNAc) and one-receptor, two-ligand binding curves (UDP). is the receptor-ligand binding free energy (kcal/mol), is the receptor efficacy, is the background intensity, and is the noise parameter which quantifies the discrepancy between the model and the observed binding curves. Due to antagonistic activity of UDP, 50/50 UDP+UDP-Glc binary mixture was used with K-3, L-3, 2211 and 50/50 UDP+UDP-Gal binary mixture was used with H-20 to predict UDP parameters (compound concentrations were set to their exact values for these calibration predictions). In each case, the mixture was chosen on the basis of the smallest standard deviation of . (PDF) [file pcbi.1002224.s014.pdf]

		UDP (L1)	UDP-Gal (L2)	UDP-Glc (L3)	UDP-GlcNAc (L4)
**H-20 (R1)**	ΔG	$-7.42 \pm .05$	$-6.69 \pm .04$	$-6.29 \pm .06$	$-6.16 \pm .06$
	A	$.008 \pm .009$	$.994 \pm .005$	$.74 \pm .01$	$.51 \pm .01$
	b	$.006 \pm .005$	$.015 \pm .009$	$.014 \pm .009$	$.008 \pm .006$
	$\tilde{\sigma}$	$.036 \pm .004$	$.041 \pm .005$	$.037 \pm .005$	$.029 \pm .004$
**K-3 (R2)**	ΔG	$-8.2 \pm .2$	$-5.64 \pm .07$	$-5.36 \pm .04$	$-5.36 \pm .06$
	A	$.18 \pm .01$	$.81 \pm .02$	$.94 \pm .02$	$.55 \pm .01$
	b	$.10 \pm .02$	$.11 \pm .01$	$.069 \pm .008$	$.111 \pm .006$
	$\tilde{\sigma}$	$.022 \pm .003$	$.043 \pm .005$	$.030 \pm .004$	$.022 \pm .003$
**L-3 (R3)**	ΔG	$-7.4 \pm .1$	$-6.21 \pm .07$	$-6.6 \pm .1$	$-5.60 \pm .06$
	A	$.02 \pm .02$	$.85 \pm .03$	$.83 \pm .03$	$.43 \pm .01$
	b	$.01 \pm .01$	$.02 \pm .01$	$.04 \pm .02$	$.007 \pm .004$
	$\tilde{\sigma}$	$.064 \pm .009$	$.049 \pm .007$	$.070 \pm .009$	$.018 \pm .002$
**2211 (R4)**	ΔG	$-10.3 \pm .3$	$-7.69 \pm .09$	$-8.48 \pm .08$	$-8.00 \pm .09$
	A	$.010 \pm .009$	$.82 \pm .04$	$.85 \pm .02$	$.89 \pm .04$
	b	$0.05 \pm .01$	$.094 \pm .008$	$.08 \pm .01$	$.11 \pm .01$
	$\tilde{\sigma}$	$.019 \pm .002$	$.035 \pm .005$	$.046 \pm .006$	$.044 \pm .006$
