# Supplementary material for: Decoding Complex Chemical Mixtures with a Physical Model of a Sensor Array
Source: PLoS Comput Biol. 2011 Oct 20;7(10):e1002224. doi: 10.1371/journal.pcbi.1002224 (PMC3202980; doi:10.1371/journal.pcbi.1002224)
Supplement: Table S2 — Prediction of ligand concentrations in equal-proportion mixtures (data for Fig. 2 ). (PDF) [file pcbi.1002224.s015.pdf]

		[L1]	[L2]	[L3]	[L4]	[Total]
**L1**	predicted	$2.8 \pm .7$	$.01 \pm .01$	$.001 \pm .001$	$.10 \pm .08$	2.9 ± 0.8
	actual	1.0	0	0	0	1.0
**L2**	predicted	$.0002 \pm .0001$	$1.05 \pm .04$	$.006 \pm .008$	$.004 \pm .009$	$1.06 \pm .03$
	actual	0	1.0	0	0	1.0
**L3**	predicted	$.0003 \pm .0002$	$.01 \pm .01$	$.96 \pm .04$	$.003 \pm .009$	$.97 \pm .04$
	actual	0	0	1.0	0	1.0
**L4**	predicted	$2 \times 10^{-4} \pm 6 \times 10^{-6}$	$.0004 \pm .0005$	$.0008 \pm .0007$	$1.05 \pm .04$	$1.05 \pm .04$
	actual	0	0	0	1.0	1.0
**L1 + L2**	predicted	$.093 \pm .007$	$.092 \pm .007$	$4 \times 10^{-5} \pm 4 \times 10^{-5}$	$.001 \pm .002$	$.19 \pm .01$
	actual	.5	.5	0	0	1.0
**L1 + L3**	predicted	$.082 \pm .009$	$.023 \pm .004$	$.09 \pm .01$	$.000 \pm .001$	$.20 \pm .02$
	actual	.5	0	.5	0	1.0
**L1 + L4**	predicted	$.48 \pm .08$	$.0001 \pm .0002$	$.001 \pm .001$	$.26 \pm .05$	0.7 ± 0.1
	actual	.5	0	0	.5	1.0
**L2 + L3**	predicted	$.006 \pm .001$	$.51 \pm .03$	$.52 \pm .03$	$.001 \pm .003$	$1.03 \pm .04$
	actual	0	.5	.5	0	1.0
**L2 + L4**	predicted	$.0002 \pm .0002$	$.58 \pm .04$	$.002 \pm .004$	$.51 \pm .04$	$1.10 \pm .06$
	actual	0	.5	0	.5	1.0
**L3 + L4**	predicted	$.0054 \pm .0008$	$.02 \pm .01$	$.62 \pm .07$	$.28 \pm .06$	$.92 \pm .06$
	actual	0	0	.5	.5	1.0
**L1 + L2 + L3**	predicted	$.049 \pm .006$	$.12 \pm .01$	$.033 \pm .008$	$.06 \pm .01$	$.26 \pm .02$
	actual	.33	.33	.33	0	1.0
**L1 + L2 + L4**	predicted	$.08 \pm .02$	$.027 \pm .006$	$1 \times 10^{-5} \pm 3 \times 10^{-5}$	$.36 \pm .04$	$.46 \pm .04$
	actual	.33	.33	0	.33	1.0
**L1 + L3 + L4**	predicted	$.064 \pm .007$	$.038 \pm .005$	$.068 \pm .007$	$.003 \pm .007$	$.17 \pm .02$
	actual	.33	0	.33	.33	1.0
**L2 + L3 + L4**	predicted	$.0049 \pm .0009$	$.25 \pm .03$	$.46 \pm .04$	$.02 \pm .02$	$.73 \pm .04$
	actual	0	.33	.33	.33	1.0
**All Four**	predicted	$.063 \pm .007$	$.20 \pm .02$	$.008 \pm .008$	$.20 \pm .02$	$.45 \pm .04$
	actual	.25	.25	.25	.25	1.0
