# Supplementary material for: Decoding Complex Chemical Mixtures with a Physical Model of a Sensor Array
Source: PLoS Comput Biol. 2011 Oct 20;7(10):e1002224. doi: 10.1371/journal.pcbi.1002224 (PMC3202980; doi:10.1371/journal.pcbi.1002224)
Supplement: Table S3 — Prediction of ligand concentrations in unequal-proportion binary mixtures of [UDP-Gal] and [UDP-Glc] (data for Fig. 3 ). (PDF) [file pcbi.1002224.s016.pdf]

[UDP-Glc]/[UDP-Gal]		α_1	α_2	α_3	$\log_{10}$[Total]
**90/10**	predicted	11 ± 9	5 ± 4	$.001 \pm .002$	$-2.87 \pm .03$
	actual	9	0	0	-3
**80/20**	predicted	$2.3 \pm .6$	0.9 ± 0.3	$.0004 \pm .0002$	$-2.89 \pm .03$
	actual	4	0	0	-3
**60/40**	predicted	$.71 \pm .09$	$.01 \pm .02$	$.0010 \pm .0007$	$-2.95 \pm .03$
	actual	1.5	0	0	-3
**40/60**	predicted	0.8 ± 0.2	$.05 \pm .09$	$.0005 \pm .0004$	$-2.91 \pm .02$
	actual	0.66	0	0	-3
**20/80**	predicted	0.7 ± 0.1	$.05 \pm .05$	$.0026 \pm .0007$	$-2.83 \pm .02$
	actual	0.25	0	0	-3
**10/90**	predicted	$.20 \pm .07$	$.01 \pm .02$	$.0031 \pm .0007$	$-2.85 \pm .03$
	actual	0.11	0	0	-3
