# Supplementary material for: Decoding Complex Chemical Mixtures with a Physical Model of a Sensor Array
Source: PLoS Comput Biol. 2011 Oct 20;7(10):e1002224. doi: 10.1371/journal.pcbi.1002224 (PMC3202980; doi:10.1371/journal.pcbi.1002224)
Supplement: Table S4 — Prediction of ligand concentrations in unequal-proportion binary mixtures of [UDP-Gal] and [UDP-Glc] using an alternative definition of relative concentrations. We used nested sampling of a four-receptor, four-ligand model to infer relative concentrations , , , as well as the total concentration [Total] = [UDP-Gal]+[UDP-Glc]+[UDP-GlcNAc]+[UDP] at the reference point. 's and 's were refit to account for “plate bias”: small systematic deviations in the values of and (from the standard values shown in Table S1 and used everywhere else) between different plates. For Plate 1 (measurements 1–3), , and were set to , and for H-20, K-3, L-3 and 2211, respectively. For Plate 2 (measurements 4–6), the corresponding values were , and . and were taken from Table S1. (PDF) [file pcbi.1002224.s017.pdf]

[UDP-Gal]/[UDP-Glc]		α_1	α_2	α_3	$\log_{10}$[Total]
**10/90**	predicted	$.48 \pm .09$	0.5 ± 0.1	$.0002 \pm .0001$	$-2.94 \pm .03$
	actual	.11	0	0	-3
**20/80**	predicted	$1.0 \pm .2$	0.4 ± 0.3	$.0003 \pm .0002$	$-2.94 \pm .04$
	actual	.25	0	0	-3
**40/60**	predicted	$3.2 \pm .4$	$.02 \pm .04$	$.003 \pm .002$	$-2.95 \pm .03$
	actual	.66	0	0	-3
**60/40**	predicted	3.3 ± 0.8	$.01 \pm .03$	$.0003 \pm .0002$	$-2.89 \pm .02$
	actual	1.5	0	0	-3
**80/20**	predicted	7 ± 2	$.02 \pm .03$	$.003 \pm .002$	$-2.80 \pm .03$
	actual	4	0	0	-3
**90/10**	predicted	54 ± 25	0.0 ± 0.1	$.03 \pm .03$	$-2.78 \pm .03$
	actual	9	0	0	-3
