# Supplementary material for: Decoding Complex Chemical Mixtures with a Physical Model of a Sensor Array
Source: PLoS Comput Biol. 2011 Oct 20;7(10):e1002224. doi: 10.1371/journal.pcbi.1002224 (PMC3202980; doi:10.1371/journal.pcbi.1002224)
Supplement: Text S1 — Supplementary Materials and Methods. (PDF) [file pcbi.1002224.s018.pdf]

Text S1

Reagents. UDP-glucose (UDP-Glc), UDP-galactose (UDP-Gal), UDP-N-acetylglucosamine (UDP-GlcNAc), uridine diphosphate (UDP) and fluorescin (FDG) were purchased from Sigma-Aldrich (St. Louis, MO).

Strains and plasmids. Mutagenesis and selection were performed in yeast strain CY10560 (P_{FUS1}-$HIS3$ $ade2\Delta3447$ $ade8\Delta3457$ $can1$-100 $far1\Delta1442$ $his3\Delta200$ $leu2$-3, 112 $lys2$ $sst2\Delta1056$ $ste14$ :: $trp1$ :: $LYS2$ $ste18\gamma6$-3841 $ste3\Delta1156$ $trp1$-1 $ura3$-52). β-galactosidase assays were performed using yeast strain CY10981 ($PFUS1$-$HIS3$ $can1$-100 $far1\Delta1442$ $his3\Delta200$ $leu2$-3, 112 $lys2$ $sst2\Delta2$ $ste14$ :: $trp1$ :: $LYS2$ $ste3\Delta1156$ $trp1$-1 $ura3$-52) carrying plasmid Cp1021 (P_{FUS1}-$LacZ$ $2\mu m$ $URA3$). The UDP-glucose receptor and 2211, H-20 and K-3 mutants were cloned as previously described.[1]

Hessian matrix as a measure of sensitivity to model parameters. Differentiating log-likelihood (Eq. (8) in the main text) twice with respect to model parameters yields:

$$\frac{\partial^2 \mathcal{L}}{\partial \gamma_i \partial \gamma_j} = -\sum_{k=1}^{N_{rec}} \frac{1}{\tilde{\sigma}_k^2} \sum_{l=1}^{N_k} \left\{ \frac{\partial I_k^l}{\partial \gamma_i} \frac{\partial I_k^l}{\partial \gamma_j} + \left[I_k^l(\{\gamma\}) - \tilde{I}_k^l \right] \frac{\partial^2 I_k^l}{\partial \gamma_i \partial \gamma_j} \right\}, \tag{1}$$

where $\{\gamma\} = (\{\alpha\}, \mu)$. The second term can be omitted in the low-noise limit ($I_k^l(\{\alpha\}, \mu) - \tilde{I}_k^l \sim \tilde{\sigma}_k \to 0$), yielding a standard expression for the Hessian matrix[2] (Eq. (9) in the main text). Explicitly,

$$\begin{aligned}
\frac{\partial^2 \mathcal{L}}{\partial \alpha_i \partial \alpha_j} &= -\sum_{k=1}^{N_{rec}} \frac{1}{\tilde{\sigma}_k^2} \sum_{l=1}^{N_k} \left(\frac{p_{i+1}^{k,l}}{\alpha_i}(A_{i+1}^k - I_k^l) - \frac{I_k^l}{\mathcal{Z}^{k,l} S} \right) \left(\frac{p_{j+1}^{k,l}}{\alpha_j}(A_{j+1}^k - I_k^l) - \frac{I_k^l}{\mathcal{Z}^{k,l} S} \right), \\
\frac{\partial^2 \mathcal{L}}{\partial \mu^2} &= -\beta^2 \sum_{k=1}^{N_{rec}} \frac{1}{\tilde{\sigma}_k^2} \sum_{l=1}^{N_k} \left(\frac{I_k^l}{\mathcal{Z}^{k,l}} \right)^2, \qquad (2) \\
\frac{\partial^2 \mathcal{L}}{\partial \mu \partial \alpha_i} &= -\beta \sum_{k=1}^{N_{rec}} \frac{1}{\tilde{\sigma}_k^2} \sum_{l=1}^{N_k} \left(\frac{I_k^l}{\mathcal{Z}^{k,l}} \right) \left(\frac{p_{i+1}^{k,l}}{\alpha_i}(A_{i+1}^k - I_k^l) - \frac{I_k^l}{\mathcal{Z}^{k,l} S} \right).
\end{aligned}$$

Hessian analysis of a mixture of two ligands interacting with a single receptor. For a single receptor and two ligands we obtain from Eq. (2) (omitting the receptor index and setting $\alpha_1 = \alpha,\ \tilde{\sigma}^2 = 1$ for convenience):

$$\begin{aligned}
\frac{\partial^2 \mathcal{L}}{\partial \alpha^2} &= -\sum_{l=1}^{N} \left(\frac{p_2^l}{\alpha}(A_2 - I^l) - \frac{I^l}{\mathcal{Z}^l(1+\alpha)} \right)^2, \qquad (3) \\
\frac{\partial^2 \mathcal{L}}{\partial \mu^2} &= -\beta^2 \sum_{l=1}^{N} \left(\frac{I^l}{\mathcal{Z}^l} \right)^2, \\
\frac{\partial^2 \mathcal{L}}{\partial \mu \partial \alpha} &= -\beta \sum_{l=1}^{N} \left(\frac{I^l}{\mathcal{Z}^l} \right) \left(\frac{p_2^l}{\alpha}(A_2 - I^l) - \frac{I^l}{\mathcal{Z}^l(1+\alpha)} \right).
\end{aligned}$$

Alternative definitions of the relative concentrations. Recall that the relative concentrations are defined as $\alpha_m = n_{m+1}/n_1$ ($m = 1 \ldots N_{lig} - 1$), where n_j is the concentration of ligand $j = 1 \ldots N_{lig}$. Different choices of the ligand in the denominator may lead to very different numerical values of α if, for example, $n_1 \ll n_2$ in a two-ligand, one-receptor system. Nevertheless, the uncertainty of both predictions is related. Indeed, if $\alpha = n_2/n_1 \to 0$ one can show that

$$\frac{\partial^2 \mathcal{L}}{\partial \alpha^2} \to -\sum_{l=1}^{N} \left(\frac{e_1^l e_2^l (A_2 - A_1) - A_1 e_1^l + A_2 e_2^l}{(1 + e_1^l)^2} \right)^2, \tag{4}$$

where $e_i^l = \exp\{-\beta(\Delta G_i - \mu^l)\}$, $i = 1, 2$. Thus the absence of ligand 2 can generally be predicted with finite uncertainty, at least if the total chemical potential μ is known. In the $\alpha \to +\infty$ limit,

$$\frac{\partial^2 \mathcal{L}}{\partial \alpha^2} \to -\frac{1}{\alpha^4} \sum_{l=1}^{N} \left(\frac{e_1^l e_2^l (A_2 - A_1) - A_1 e_1^l + A_2 e_2^l}{(1 + e_2^l)^2} \right)^2, \tag{5}$$

and $\sigma_\alpha^2 = -\left(\partial^2 \mathcal{L}/\partial \alpha^2\right)^{-1}$ diverges as α^4. This is expected because $\alpha \to +\infty$ is equivalent to $\alpha' = n_1/n_2 = 1/\alpha \to 0$, yielding $\sigma_\alpha^2 = \alpha^4 \sigma_{\alpha'}^2$. Thus $\sigma_{\alpha'}^2$ remains finite as ligand 1 disappears from the mixture. Moreover, the expression for $\partial^2 \mathcal{L}/\partial \alpha'^2$ in the $\alpha' \to 0$ limit should be the same as the expression for $\partial^2 \mathcal{L}/\partial \alpha^2$ in the $\alpha \to 0$ limit, but with ligand labels 1 and 2 interchanged. Indeed, if $1 \leftrightarrow 2$ Eq. (5) becomes the same as Eq. (4), apart from the $1/\alpha^4$ factor mentioned above.
Agonist-agonist scenario. If both ligands have unit efficacies ($A_1 = A_2 = 1$), Eq. (3) gives

$$\frac{\partial^2 \mathcal{L}}{\partial \alpha^2} = -\sum_{l=1}^{N} \left(\frac{p_2^l - \alpha p_1^l}{\mathcal{Z}^l \alpha (1 + \alpha)} \right)^2. \tag{6}$$

Let us assume for simplicity that μ is known, so that $\sigma_\alpha^2 = -\left(\partial^2 \mathcal{L}/\partial \alpha^2\right)^{-1}$. Furthermore, let us suppose that ΔG_1 is fixed at a finite value, while ΔG_2 varies from $-\infty$ to $+\infty$. It is then easy to see that $\Delta G_2 = \Delta G_1$ is a special case, yielding $p_2^l = \alpha p_1^l$ ($\forall l$) and thus $\sigma_\alpha^2 = \infty$. So, as expected, discrimination between the two ligands is impossible if they have equal efficacies and binding affinities. If $\Delta G_2 \to -\infty$, $\mathcal{Z}^l \to +\infty$ (and $p_2^l \to 1, \forall l$), making $\partial^2 \mathcal{L}/\partial \alpha^2 = 0$ for finite α. Thus discrimination is impossible if one of the ligands completely saturates the receptor. However, if $\Delta G_2 \to +\infty$, $\mathcal{Z}^l$ remains finite (while $p_2^l \to 0, \forall l$), yielding

$$\frac{\partial^2 \mathcal{L}}{\partial \alpha^2} \simeq -\frac{1}{(1 + \alpha)^2} \sum_{l=1}^{N} \left(\frac{p_1^l}{\mathcal{Z}^l} \right)^2. \tag{7}$$

Surprisingly, discrimination is still possible in this limit, even if ligand 2 does not bind the receptor (**Fig. S9**). This is because the total concentration is known in this example and so the information provided by ligand 1 is sufficient to infer α. Of course, if ligand

1 is either unbound ($p_1^l \to 0, \forall l$) or strongly bound ($\mathcal{Z}^l \to +\infty$), predicting α becomes impossible again.

If α is known, the error in the predicted total chemical potential μ is determined by (Eq. (3)):

$$\frac{\partial^2 \mathcal{L}}{\partial \mu^2} = -\beta^2 \sum_{l=1}^{N} \left(\frac{\mathcal{Z}^l - 1}{\mathcal{Z}^{l2}} \right)^2 . \tag{8}$$

Learning the value of the total concentration becomes impossible if both ligands are unbound ($\mathcal{Z}^l \to 1$), or if one ligand is bound so strongly that measuring the concentration of the other ligand becomes problematic ($\mathcal{Z}^l \to \infty$). Note that in both of these limits $\partial^2\mathcal{L}/\partial\alpha^2 = 0$ as well.

Simultaneous discrimination of μ and α is not possible if both ligands have equal efficacies (**Fig. S9**). Indeed, the determinant of the 2×2 matrix of second derivatives is always close to 0:

$$\begin{aligned} \det\left(\frac{\partial^2 \mathcal{L}}{\partial \gamma_i \partial \gamma_j} \right) &= \left(\frac{\beta}{\alpha(1+\alpha)} \right)^2 \sum_{l,l'=1}^{N} \frac{\mathcal{Z}^{l'} - 1}{\mathcal{Z}^{l2} \mathcal{Z}^{l'3}} (p_2^l - \alpha p_1^l) \times \\ & \left[(p_2^l - \alpha p_1^l)(p_1^{l'} + p_2^{l'}) - (p_2^{l'} - \alpha p_1^{l'})(p_1^l + p_2^l) \right] . \end{aligned} \tag{9}$$

This is because appreciable values of p_1^l or p_2^l lead to $\mathcal{Z}^l > 1$, which in turn suppresses the determinant. The small value of the determinant means that at least one of the errors is large. For example, if $\Delta G_2 = \Delta G_1$,

$$\sigma_\alpha^2 = -\frac{1}{\det\left(\frac{\partial^2 \mathcal{L}}{\partial \gamma_i \partial \gamma_j} \right)} \frac{\partial^2 \mathcal{L}}{\partial \mu^2} \tag{10}$$

is infinite because $p_2^l = \alpha p_1^l (\forall l)$, whereas $\mathcal{Z}^l$ and thus $\partial^2\mathcal{L}/\partial\mu^2$ are finite. In general, the multidimensional analysis of this type is difficult because zeros in the numerator and denominator of Eq. (10) and a similar equation for σ_μ^2 have to be handled correctly.

Agonist-antagonist and antagonist-agonist scenarios. If one of the ligands (*e.g.* ligand 2) acts as a perfect antagonist ($A_1 = 1, A_2 = 0$), Eq. (3) gives (**Fig. S8**):

$$\begin{aligned} \frac{\partial^2 \mathcal{L}}{\partial \alpha^2} &= -\sum_{l=1}^{N} (p_1^l)^2 \left(\frac{p_2^l}{\alpha} + \frac{1}{\mathcal{Z}^l (1+\alpha)} \right)^2 , \\ \frac{\partial^2 \mathcal{L}}{\partial \mu^2} &= -\beta^2 \sum_{l=1}^{N} \left(\frac{p_1^l}{\mathcal{Z}^l} \right)^2 , \\ \frac{\partial^2 \mathcal{L}}{\partial \mu \partial \alpha} &= -\beta \sum_{l=1}^{N} \left(\frac{{p_1^l}^2}{\mathcal{Z}^l} \right) \left(\frac{p_2^l}{\alpha} + \frac{1}{\mathcal{Z}^l (1+\alpha)} \right) . \end{aligned} \tag{11}$$

With ligand 1 acting as an antagonist ($A_1 = 0, A_2 = 1$), we obtain (**Fig. S7**):

$$
\begin{aligned}
\frac{\partial^2 \mathcal{L}}{\partial \alpha^2} &= -\sum_{l=1}^{N} \left(p_2^l\right)^2 \left(\frac{p_1^l}{\alpha} + \frac{1}{\mathcal{Z}^l \alpha(1+\alpha)}\right)^2, \\
\frac{\partial^2 \mathcal{L}}{\partial \mu^2} &= -\beta^2 \sum_{l=1}^{N} \left(\frac{p_2^l}{\mathcal{Z}^l}\right)^2, \\
\frac{\partial^2 \mathcal{L}}{\partial \mu \partial \alpha} &= -\beta \sum_{l=1}^{N} \left(\frac{{p_2^l}^2}{\mathcal{Z}^l}\right) \left(\frac{p_1^l}{\alpha} + \frac{1}{\mathcal{Z}^l \alpha(1+\alpha)}\right).
\end{aligned}
\tag{12}
$$

If $\alpha = 1$ Eq. (12) is the same as Eq. (11) with ΔG_1 and ΔG_2 interchanged. However, for arbitrary α there is no symmetry, so that maximizing the determinant of the Hessian with $A_1 = 0, A_2 = 1$ and $A_1 = 1, A_2 = 0$ yields two distinct solutions in the $\{\Delta G_1, \Delta G_2\}$ space.

Number of agonist-antagonist patterns in an arbitrary receptor array. For an arbitrary receptor-ligand combination, there are

$$
\sum_{m=1}^{N_{rec}-1} \frac{(m+1)!}{N_{rec}!} n^{N_{rec}-m} + \frac{n(n^{N_{rec}} - 2n^{N_{rec}-1} + 1)}{(n-1)N_{rec}!}
\tag{13}
$$

unique patterns in which each receptor interacts with one agonist and one antagonist (the patterns are unique in a sense that all patterns connected by trivial receptor label permutations are counted only once). Here N_{rec} is the number of receptors, N_{lig} is the number of ligands, and $n = 2\binom{N_{lig}}{2}$ is the number of ways in which one agonist and one antagonist can be bound by a single receptor, so that the total number of patterns (some connected by receptor label permutations) is $n^{N_{rec}}$. With $N_{rec} = 2$ and $N_{lig} = 3$ the total number of patterns is 36, and 21 unique patterns include 3 combinations in which one of the ligands acts as an antagonist for both receptors, 3 more where one of the ligands is the global agonist, 6 mixed patterns where one ligand invokes the agonist-antagonist receptor response and the other two are either an agonist or an antagonist for one of the remaining receptors, and finally 9 patterns in which one of the receptors does not strongly interact with any of the ligands. The remaining 15 patterns are related to the ones listed above through receptor label permutations. The first subclass yields equivalent globally optimal solutions, subclasses 2 and 3 correspond to local maxima, and the fourth subclass in which one of the receptors remains unused does not yield any stable solutions, relaxing into one of the other categories.

References

[1] Ault A, Broach J (2006) Creation of GPCR-based chemical sensors by directed evolution in yeast. Prot Eng Des Sel 19: 1-8.

[2] Gutenkunst R, Waterfall J, Casey F, Brown K, Myers C, et al. (2007) Universally sloppy parameter sensitivities in systems biology models. PLoS Comp Biol 3: 1871-1878.
